# Herb-drug interactions of silybinin and cilofexor in beagle dogs based on pharmacokinetics by UPLC-MS/MS

**DOI:** 10.3389/fphar.2024.1334402

**Published:** 2024-02-08

**Authors:** Xinyi Wei, Yanding Su, Qian Cheng, Songmao Liang, Tingping Zhang, Lengxin Duan, Xiuwei Shen, Xiangjun Qiu

**Affiliations:** ^1^ College of Basic Medicine and Forensic Medicine, Henan University of Science and Technology, Luoyang, Henan, China; ^2^ Ruian People’s Hospital, The Third Affiliated Hospital of Wenzhou Medical University, Wenzhou, Zhejiang, China

**Keywords:** silybinin, cilofexor, UPLC-MS/MS, pharmacokinetics, HDIS, beagle dogs

## Abstract

**Objective:** A remarkably sensitive, accurate, and efficient ultra-performance liquid chromatography-tandem mass spectrometry (UPLC-MS/MS) approach was developed as a facile and expeditious method for measuring cilofexor concentration in beagle dogs, the herb-drug interactions between silybinin and cilofexor was explored based on pharmacokinetics.

**Methods:** The plasma sample protein of the beagles were rapidly sedimented with acetonitrile, and cilofexor and tropifexor (internal standard, ISTD) were separated by gradient elution using a 0.1% formic acid aqueous solution and acetonitrile as the mobile phase. The concentrations were detected using positive ion multiple reaction monitoring (MRM) mode. Mass transfer pairs were m/z 587.91→267.91 for cilofexor and m/z 604.08→228.03 for ISTD, respectively. A two-period self-controlled experimental design was adopted for the HDIs experiment. In the first period (Group A), six beagle dogs were orally administered cilofexor at a dose of 1 mg/kg. In the second period (Group B), silybinin (3 mg/kg) was orally administered to the six beagle dogs twice a day for seven consecutive days, after which cilofexor was orally administered. The cilofexor concentration in beagle dogs was determined, and HDIs were evaluated based on their pharmacokinetics.

**Results:** The accuracy and precision of cilofexor were both less than 15%, and the recoveries, matrix effects, and stability met the relevant requirements. The C_max_ of cilofexor in group B was 49.62% higher than that in group A, whereas the AUC_(0-t)_ and AUC_(0−∞)_ of cilofexor in group B were 47.85% and 48.52% higher, respectively, than those in group A. Meanwhile, the t_1/2_ extended from 7.84 h to 9.45 h, CL and Vz decreased in Group B.

**Conclusion:** A novel UPLC-MS/MS approach was successfully applied for the measurement of cilofexor in beagle dog plasma. Silybinin can alter the pharmacokinetics of cilofexor in beagle dogs, thereby increasing plasma exposure to cilofexor.

## Introduction

Non-alcoholic steatohepatitis (NASH) is an inflammatory subtype of non-alcoholic fatty liver disease (NAFLD) that is associated with disease progression, cirrhosis development, and liver transplant needs. Although NASH is important, it is underestimated in clinical practice (Sheka et al., 2020). NASH is a subtype of NAFLD that can develop into cirrhosis, hepatocellular carcinoma (HCC), and death NAFLD and NASH are not only found in adults but also in children and adolescents with a high incidence rate of these diseases (Younossi et al., 2019). NASH is closely related to obesity, dyslipidemia, type 2 diabetes, and metabolic syndrome. The latest model predicts that the incidence rates of NAFLD and NASH will increase, causing a huge clinical and economic burden, and the results reported by patients are poor (Younossi et al., 2019; Sheka et al., 2020). It is predicted that NASH-related liver deaths will increase by 178% by 2030 (Younossi et al., 2019). Therefore, this is an important stage in the progression of simple fatty liver to liver fibrosis, cirrhosis, and cancer.

The mechanism of NASH progression remains unclear and there is a lack of effective treatment methods. Therefore, it is necessary to understand the pathogenic mechanisms that lead to the development and progression of diseases to develop innovative therapies (Leng et al., 2021). While there are no FDA-approved medications for NAFLD/NASH, dietary and lifestyle interventions are the mainstay of treatment, and pharmacological therapy also plays a certain role in the treatment of NASH, including insulin sensitizers, antioxidants, lipotoxicity-based targets, modulation of nuclear transcription factors, gastrointestinal hormones, and cytokines. Several medications are in the pipeline of therapy for NASH and hold promise for successful therapy in the future (Raza et al., 2021).

FXR exerts anti-inflammatory effects through SHP-dependent and independent pathways. The binding of FXR to ligands reduces inflammation via induction of SHP expression, stabilization of chemokine 2 (CCL2) promoter inhibitor complex, or direct promotion of nuclear receptor co-inhibitor 1 (NCor1) complex binding to pro-inflammatory gene promoters and downregulation of pro-inflammatory factor expression (Zou et al., 2018). One study has demonstrated that activation of hepatic FXR improves the histological features of NASH by inducing the expression of fibroblast growth factor 21 (FGF21), a strong regulator of lipocalin, via the induction of upregulation of lipocalin expression (Cyphert et al., 2012), thereby improving insulin resistance and hepatic fat accumulation (Liu et al., 2020). Another study suggested that FGF21 inhibits the progression of NASH to hepatocellular carcinoma through inhibition of Toll-like receptors and interleukin-17 signaling in hepatocytes.

**FIGURE 1:**
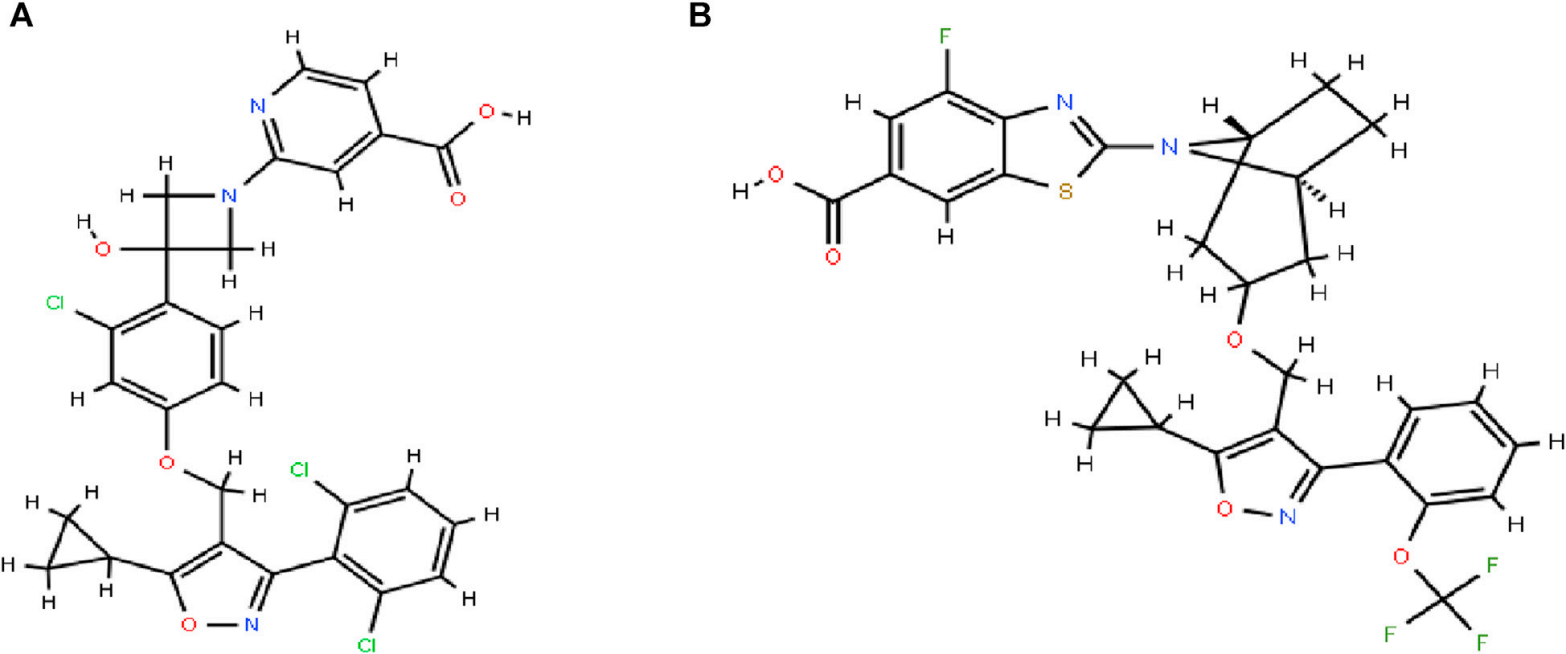
The chemical construction of cilofexor **(A)** and tropifexor **(B)**.

Cilofexor (GS-9674, Figure 1A) was successfully researched and developed as a selective, non-steroidal FXR agonist by Gilead Sciences (Trauner et al., 2019). FXR affects liver metabolism, inflammation, and liver fibrosis as a key component of NASH, and reduces portal hypertension and liver fibrosis in NASH rats (Schwabl et al., 2021). Cilofexor was well tolerated and significantly reduced hepatic steatosis (median relative decrease in magnetic resonance imaging proton density fat fraction [MRI-PDFF] of −22.7% in those receiving 100 mg of cilofexor compared with an increase of 1.9% in those receiving placebo) (Patel et al., 2020). Cilofexor at a dose of 100 mg was associated with moderate to severe pruritus in 14% of patients (4% in the placebo group). Cilofexor was well tolerated and improved markers of cholestasis, liver biochemistry, C4, and serum bile acids in patients with primary sclerosing cholangitis (Schwabl et al., 2021).

The primary pathway for the elimination of cilofexors is hepatic metabolism, and the major metabolites are GS-716070 and GS-1056756. Cilofexor is a substrate of cytochrome P450 isoforms 2C8 and 2C19 as well as a substrate for human efflux transporters, P-glycoprotein, breast cancer resistance protein, uptake transporters, and organic anion transporting polypeptides (OATPs; OATP1B1, OATP1B3, and OATP2B1). (Trauner et al., 2019).

Traditional Chinese medicines (TCMs) are abundant sources of biologically active substances that can be used to prevent human diseases. Currently, an increasing number of studies have focused on natural products, which have shown potential effects against NAFLD (Zhou et al., 2020). The natural constituents have been categorized into four subsets according to their functions: regulating metabolism, regulating inflammation, regulating fibrosis, and regulating intestinal microbiota. A deeper exploration of natural compounds may be a promising direction for curing NASH (Leng et al., 2021).

Silybinin is a flavonoid isolated from the fruits and seeds of milk thistle of the Asteraceae family and has a wide range of pharmacological activities such as hepatoprotective, antioxidant, antitumor activity, and maintenance of hepatic cell membrane stability. Silybinin is regarded as the most potent flavonoid against liver disease and can prevent NASH through activation of CFLAR expression and inhibition of JNK phosphorylation, which can modulate NASH-associated liver lipid metabolism, insulin resistance, and oxidative stress (Liu et al., 2019). Silybinin may reduce hepatic damage in methotrexate-induced hepatotoxicity in rats by increasing antioxidant capacity (Yanaşoğlu et al., 2022). The inflammation-reducing effect of silybinin is attributed to the inhibition of the O-GlcNAcylation-dependent NF-κB-signaling pathway. Thus, silybinin is a promising therapeutic agent for hyper-O-GlcNAcylation as well as NASH (Lee et al., 2018). Silybinin improves lipid metabolism by reducing the expression of miR-122 and inhibiting the expression of miR-122, which may be a novel therapeutic target for ameliorating fatty liver disease (Yang et al., 2021).

*In vitro* incubation showed that silybinin had a weak to moderate inhibitory effect on most CYP enzymes. Silybinin can inhibit CYP1A2, 2B6, 2C8, 2C9, 2C19, 2D6, and 3A4, but the most significant inhibitory effects were on CYP3A4 and CYP2C9 (Xie et al., 2019). Because Cilofexor is the substrate of cytochrome P450, and when used in combination with silybinin, it may cause herb-drug interactions (HDIs).

The pharmacokinetics and tolerability of Firsocostat and Cilofexor were evaluated in a phase 1 trial in participants with severe renal impairment (SRI) and healthy controls (HMCs). In the HMCs group, Cilofexor was rapidly absorbed, whereas in the SRI group, its Tmax was longer and Cmax slightly lower. AUC_last_ and AUC_inf_ of Cilofexor were similar between participants with SRI and HMCs (Weber et al., 2023). In another report it was demonstrated that a plateau in intestinal FXR activation was reached at Cilofexor doses greater than or equal to 30 mg (showing good safety and tolerability between doses of 10–300 mg). (Younis et al., 2023).

Silybinin was usually used for the treatment of liver injury, while Cilofexor was used for the treatment of NASH, and both may be co-administered in the same field, leading to herbal drug interactions. There are no reports on the interaction between the two, therefore, in the present study, the concentration of cilofexor in plasma was determined by establishing a convenient, fast, and accurate UPLC-MS/MS technique using tropifexor (Figure 1B) as an internal standard (ISTD) and the HDI between silybinin and cilofexor was explored according to the pharmacokinetics. This not only enables to correlation of the data from the pharmacological test with clinical efficacy but also helps to design a rational dosing regimen for clinical reference.

## Materials and methods

### Chemicals materials and drugs

Acetonitrile and methanol were obtained from Merck (Darmstadt, Germany) and were HPLC grade. Ultrapure water was filtered using a Milli-Q reagent system (Millipore, Billerica, MA, USA). Cilofexor (purity >98%) and tropifexor (purity >98%) were purchased from Shanghai Tronsai Technology Co., Ltd. (Shanghai, China). Silybinin Capsules (each containing 35 mg of silybinin, 250711086) were produced by Tianjin Tianshili Shengte Pharmaceutical Co. Ltd.

### Solutions preparation

Cilofexor and ISTD were both weighed 10 mg and the volume was fixed in 10 mL volumetric flasks to obtain 1 mg/mL of each standard stock solution, which was then diluted gradually with chromatographic grade methanol to obtain cilofexor working solution at concentrations of 10 μg/mL, 1 μg/mL, and 100 ng/mL as well as ISTD working solution of 1 μg/mL. The plasma standard curves were obtained by pipetting 10 μL, 50 μL of 100 ng/mL working solution, 10 μL, 25 μL, 50 μL of 1 μg/mL working solution, and 10 μL, 25 μL, 50 μL of 10 μg/mL working solution, respectively, and adding the blank plasma and then finally quantified into a 1 mL solution, which was corresponding to the concentrations of 1, 5, 10, 25, 50, 100, 250, and 500 ng/mL.

Finally, blank samples and different concentrations of cilofexor working solutions were precisely aspirated and configured into quality control (QC) samples at concentrations of 2.5, 100, and 375 ng/mL. All above-configured solutions were stored at 4°C before further experimental studies.

### Apparatus and conditions

A Waters ACQUITY UPLC instrument was used for the chromatographic analysis. Chromatographic separation was performed on a Waters ACQUITY UPLC BEH C18 column (50 mm × 2.1 mm, 1.7 μm) and a pre-column. Then, The temperature of the column chamber was maintained at 40°C, the autosampler (FTN) was set at 10°C, the temperature of the sample chamber was 4°C, the injection volume was 2 μL, and the flow rate was constant at 0.3 mL/min. Gradient elution was performed using 0.1% formic acid and acetonitrile as the mobile phases; the gradient elution procedure is presented in Table 1.

**TABLE 1:** UPLC gradient elution procedures.

Time (min)	Flow rate (mL/min)	0.1% formic (%)	Acetonitrile (%)
0	0.3	90	10
0.5	0.3	90	10
1.0	0.3	10	90
1.4	0.3	10	90
1.5	0.3	90	10
3.0	0.3	90	10

Finally, a positive ion scan was performed in multiple reaction-monitoring (MRM) acquisition modes in combination with a Waters Xevo TQ-S triple quadrupole tandem mass spectrometer (Milford, MA, USA) and an electrospray ionization source (ESI). The parameters and data of the MS/MS system acquired using Masslynx 4.1 software (Milford, Massachusetts, USA) are presented in Table 2.

**TABLE 2:** Mass spectral parameters of Cilofexor and ISTD.

Analyte	ESI	Parent ion	Daughter ion	CV (V)	CE (eV)	RT (min)
Cilofexor	+	587.91	267.91	20	30	1.57
ISTD	+	604.08	228.03	20	35	1.95

### Sample preparation

A 100 μL plasma sample was precisely dosed in a 1.5 mL EP tube, followed by the addition of 20 μL of 1 μg/mL ISTD working solution, and 300 μL acetonitrile was added to precipitate the plasma protein. Then These were mixed and vortexed together for 60 s, followed by centrifugation for 15 min (12,000 xg, 4°C). Finally, 2 μL of supernatant was pipetted for analysis.

### Methodological validation

The specificity, standard curve, accuracy, precision, stability, and matrix effect of the proposed approach were verified according to the “guiding principles for the validation of quantitative analysis methods for biological samples” in the Chinese Pharmacopoeia, 2020 edition.

Selectivity should be demonstrated using at least six batches of mixed blank beagle dog plasma. When the response of the interfering component was below 20% of the LLOQ of the analyte and below 5% of the internal standard response, it was considered acceptable.

Accuracy (RE,%) and precision (RSD,%) were assessed by preparing cilofexor QC samples (LQC 2.5 ng/mL, MQC 100 ng/mL, and HQC 375 ng/mL) and LLOQ with six identical parallel samples prepared for each concentration. The intraday precision and accuracy were calculated for 1 day of analysis, and the interday precision and accuracy were calculated for three consecutive days.

Six parallel samples were prepared by preparing cilofexor QC samples (LQC 2.5 ng/mL, MQC 100 ng/mL; HQC, 375 ng/mL) for each concentration, after which the samples were precipitated with acetonitrile and centrifuged, and the supernatant was collected for analysis. The recoveries were obtained by comparing the measured peak area ratios of cilofexor and ISTD with the area ratios of the peaks of the corresponding concentrations of standard plasma samples processed normally. The above steps were repeated by replacing the blank plasma with ultrapure water, and the measured peak area ratios of the cilofexor and ISTD were compared with those of the QC working solution samples to obtain the matrix effect.

The short-term, long-term, freeze-thaw, and autosampler stability of cilofexor were evaluated by repeatedly preparing six parallel samples of 2.5, 100, and 375 ng/mL cilofexor QC under the following four storage conditions:1) samples were left for 24 h at room temperature. 2) Store for 4 weeks at −20°C. 3) Freeze-thawing of the samples was repeated three times. 4) The samples were extracted from the sample manager (4°C) for 12 h.

### Stock solution stability

The stability of stock solutions of cilofexor and ISTD (10 μg/mL, 1 μg/mL) at room temperature and −20°C is investigated under six parallel replicate experiments. Room temperature stability was assessed by comparing a fraction of the stock solution stored at room temperature for 24 h with the remainder of the stock solution stored in a −20°C refrigerator. Freezing stability was assessed by comparing a newly configured stock solution with a stock solution stored in a −20°C refrigerator for 3 months. The evaluation of solution stability was based on the precision and accuracy of the results obtained from the tests being within a reasonable range (RE% ≤ ±10%, RSD% ≤15%).

### Animal experiments

Six beagles (7.5 _~_ 9.5 kg) were purchased from Hubei Yizhicheng Biotechnology Co., Ltd. (Yingcheng, HUBEI). The animal license number is SCXK (HUBEI) 2021-0020. Six beagle dogs were raised at the Laboratory Animal Center of Henan University of Science and Technology (Luoyang, China) and provided a normal diet and water for 1 week before the experiment. This study was approved by the Ethics Committee of the Animal Laboratory of Henan University of Science and Technology and followed the Guide for Ethical Review of Laboratory Animal Welfare (GB/T35892-2018).

The HDIs experiment utilized a two-phase self-contained experimental design. In the first period (named Group A), six beagle dogs were orally administered with cilofexor at a dose of 1 mg/kg, followed by a collection of approximately 1.0 mL of venous blood in 1.5 mL EP tubes containing heparin at 0.5, 1, 1.5, 2, 3, 4, 6, 8, 12, 24, and 48 h. Subsequently, the blood was centrifuged at 4°C for 10 min at 3,000 rpm. The supernatant was removed and stored at −20°C for subsequent analysis.

After a week of drug washout, the second period (Group B) of the experiment was performed. Six beagle dogs were orally administered silybinin 3 mg/kg twice daily for seven consecutive days. On the morning of the eighth day, beagle dogs were orally administered 1 mg/kg cilofexor 30 min after silybinin administration. Then approximately 1.0 mL of venous blood in 1.5 mL EP tubes containing heparin at 0.5, 1, 1.5, 2, 3, 4, 6, 8, 12, 24, and 48 h. The plasma was separated for subsequent analysis.

### Data analysis

The DAS 2.0 program was employed to analyze plasma cilofexor concentrations in groups A and B, and the statistical moment model was used to calculate the main pharmacokinetic parameters of cilofexor. An independent-sample *t*-test was used to compare differences in pharmacokinetic parameters between groups A and B.

## Results

### Methodological validation

#### Selectivity

**FIGURE 2:**
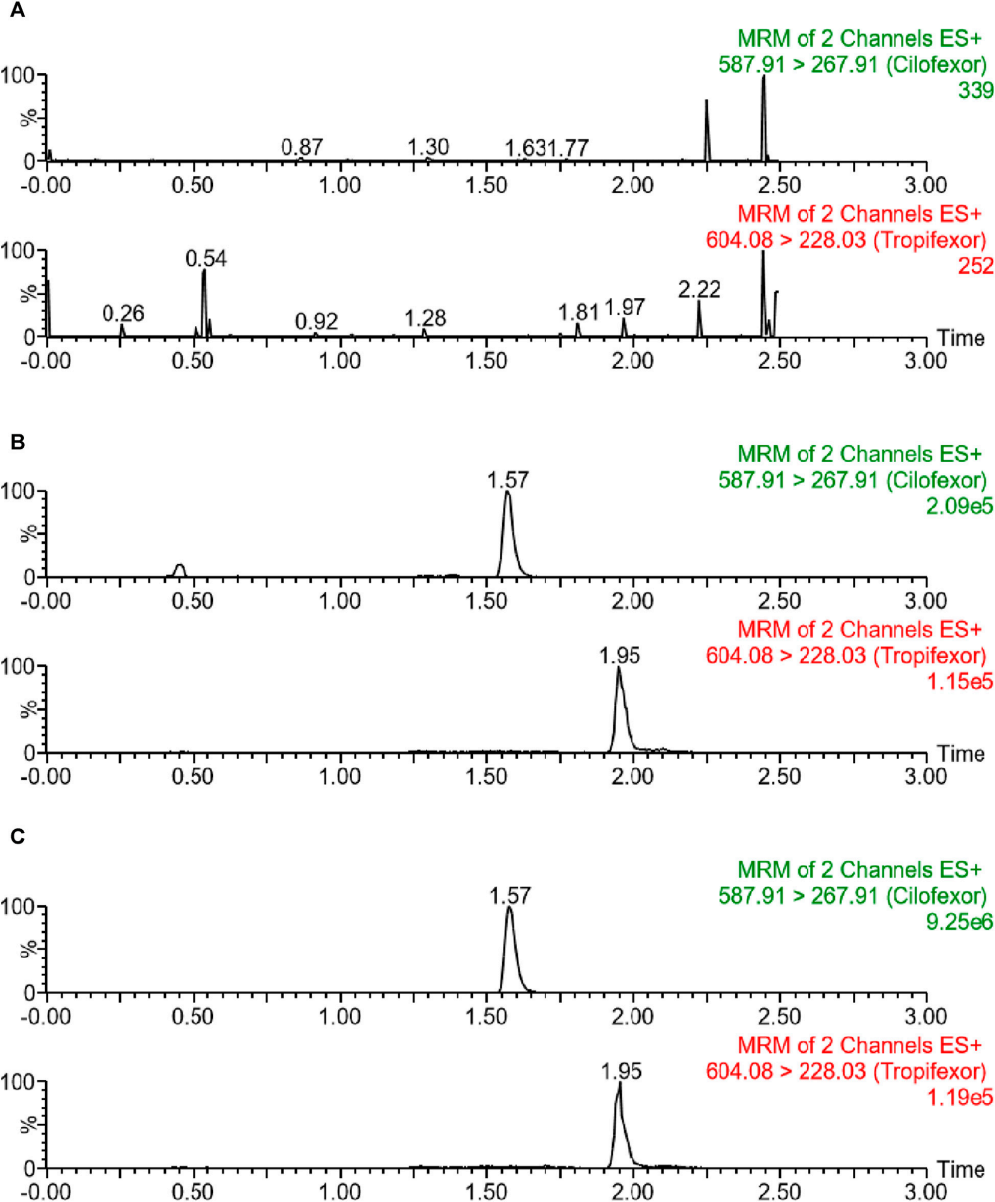
Typical chromatograms of blank plasma **(A)**, blank plasma containing 25 ng/mL cilofexor and ISTD **(B)**, as well as plasma sample following oral dosing with cilofexor **(C)**: sample 2 h after administration at 227.59 ng/mL).

In this study, the selectivity of the protocol was verified through chromatograms of three beagle plasma samples: a blank plasma specimen from a beagle, a plasma specimen with a standard solution added, and a plasma specimen obtained from a beagle following oral dosing with cilofexor. As illustrated in Figure 2, the peaks of cilofexor and ISTD were well-separated and free of endogenous interference. The retention time at cilofexor and ISTD was 1.57 min and 1.95 min, respectively.

### Limit of quantification and standard curve

The linear regression operation was performed in this study with the ratio of cilofexor to ISTD peak area on the *y*-axis, the concentration of cilofexor in plasma on the *x*-axis, and 1/X as the weighting factor, and the return equation of the linear equation was obtained as *y* = 0.0074 *x* + 0.0444 (*r* ^2^ = 0.999 3). This indicated that cilofexor showed good linearity within the 1.0–500 ng/mL, and its LLOQ value was 1.0 ng/mL.

### Accuracy and precision

As presented in Table 3, this experiment analyzed the accuracy and precision of cilofexor through multiple replicate assays on LLOQ, low-, medium-, and high-QC samples (1.0 ng/mL, 2.5 ng/mL, 100 ng/mL, 375 ng/mL). The obtained accuracy and precision ranges were within ±10%. The results demonstrated that the experimental conditions were stable for the determination of cilofexor using this method, and fulfilled the demands of analytical determination.

**TABLE 3:** Accuracy and precision in Beagle dogs plasma for cilofexor (n = 6).

Added (ng/mL)	Intra-day			Inter-day		
	Found (ng/mL)	RSD (%)	RE (%)	Found (ng/mL)	RSD (%)	RE (%)
1.0	1.02 ± 0.05	4.57	2.17	1.01 ± 0.04	4.13	0.83
2.5	2.41 ± 0.22	9.25	−3.53	2.51 ± 0.21	8.16	0.58
100	98.85 ± 6.27	6.35	−1.15	101.0 ± 74.86	4.81	1.07
375	377.12 ± 13.92	3.69	0.56	372.80 ± 15.75	4.22	−0.59

### Matrix effect and recovery

Table 4 displays the results of the recovery and ME tests, which indicated a recovery range of more than 87% for cilofexor and close to 100% for ME at 2.5, 100, and 375 ng/mL. This indicated high reproducibility and no obvious matrix effect for the cilofexors using this approach.

**TABLE 4:** Matrix effect and recovery of cilofexor in beagle dogs plasma (n = 6).

Added (ng/mL)	Recovery (%)		Matrix effect (%)	
	Mean ± SD	RSD (%)	Mean ± SD	RSD (%)
2.5	87.38 ± 4.04	4.62	99.98 ± 1.07	1.07
100	89.09 ± 2.20	2.47	99.66 ± 0.67	0.68
375	90.35 ± 1.84	2.03	100.29 ± 1.26	1.26

### Stability

The test specimens were examined and analyzed for stability at three different concentrations (2.5, 100, and 375 ng/mL). The results were stable under the four stock conditions listed in Table 5. The RSD of the cilofexor was <10%.

**TABLE 5:** The stability of cilofexor in beagle dogs plasma (n = 6).

Added (ng/mL)	Room temperature, 24 h		Autosampler 4°C, 8 h		Three freeze-thaw		−20°C, 4 weeks	
	RSD (%)	RE (%)	RSD (%)	RE (%)	RSD (%)	RE (%)	RSD (%)	RE (%)
2.5	8.32	−2.07	8.21	−2.60	7.23	−2.93	4.08	−4.53
100	6.12	−1.14	6.32	−1.01	6.32	−1.01	4.96	−2.04
375	3.11	−0.01	2.89	−0.31	2.84	−0.51	2.37	−0.93

### Stock solution stability

The stability of the stock solutions under the present experimental conditions is illustrated in Table 6. From the experimental findings, it was evident that the stock solutions of cilofexor and ISTD were stable.

**TABLE 6:** The Stock Solution Stability of cilofexor and ISTD in Beagle Plasma (n = 6).

Compounds	Spiked (µg/mL)	Room Temperature,12 h		−20°C, 3 weeks	
		RSD (%)	RE (%)	RSD (%)	RE (%)
cilofexor	10	9.68	−1.79	5.34	0.75
ISTD	1	5.32	−2.50	2.14	−1.17

## The pharmacokinetics of cilofexor

**FIGURE 3:**
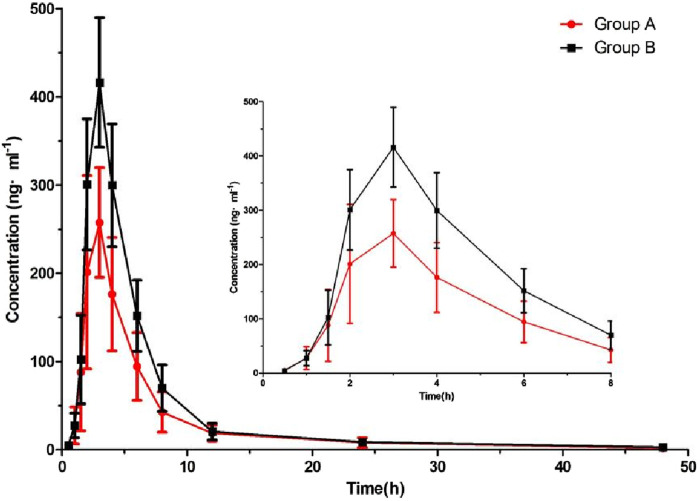
Plasma concentration-time of cilofexor in group A and group B following oral dosing with 1 mg/kg administration to beagle dogs (n = 6).

After six beagle dogs were orally administered 1 mg/kg cilofexor, the plasma mean drug concentration-time curves of cilofexor in group A and group B were shown in Figure 3, and the main pharmacokinetic parameters of cilofexor in group A and group B were listed in Table 7.

**TABLE 7:** Main pharmacokinetic parameters of cilofexor in group A and group B following oral dosing with 1 mg/kg administration to beagle dogs (n = 6, mean ± SD).

Parameters	Group A	Group B
C_max_ (ng/mL)	284.33 ± 69.26	425.41 ± 67.04**
T_max_ (h)	2.67 ± 0.52	2.83 ± 0.41
t_1/2_ (h)	7.84 ± 3.26	9.45 ± 4.54
CLz/F (L/h)	0.80 ± 0.29	0.51 ± 0.12*
Vz/F (L/kg)	9.21 ± 5.44	7.05 ± 4.64
AUC_0→*t*_ (ng·h/mL)	1,372.89 ± 487.83	2029.82 ± 449.99*
AUC_0→∞_(ng·h/mL)	1,388.65 ± 489.15	2062.48 ± 463.30*

Notes: *Compared with the group A, the difference was statistically significant (*p* < 0.05). **Compared with the group A, the difference was statistically significant (*p* < 0.01).

## The HDIs of silybinin and cilofexor

From Table 7, the results were found that the C_max_ of cilofexor in group B was 49.62% higher than that in group A, and the AUC_(0-t)_ and AUC_(0−∞)_ of cilofexor in group B were 47.85% and 48.52% higher than those in group A, respectively. At the same time, the t_1/2_ was prolonged from 7.84 h to 9.45 h, CL and Vz decreased in group B. The results showed that silybinin could slow down cilofexor metabolism and increase the plasma concentration of cilofexor in beagle dogs.

## Discussions

### Improvement of methodology

The standard mixture of cilofexor and ISTD was injected with a peristaltic pump at 400 ng/mL, and a full scan was performed in ESI+ and ESI − modes. These findings revealed that cilofexor and ISTD had better peak patterns and higher ESI + responses. This may be related to the higher stability and sensitivity of the [M + H]+ ions in the compounds. Therefore, the ESI + acquisition mode was adopted in this experiment.

In the organic mobile phase, the effects of acetonitrile and methanol on the response value and resolution of the tested components were compared. The results demonstrated that acetonitrile had a higher response and better peak shape and resolution than methanol. Therefore, acetonitrile was selected as the optimal organic phase in this study. The effects of water-acetonitrile, 0.1% acetic acid aqueous solution (acetonitrile), and 0.1% formic acid aqueous solution (acetonitrile) on the responses of the target compounds were compared, which suggested that the response was both high and specific when 0.1% formic acid aqueous solution-acetonitrile was used.

In the present study, methanol precipitation and acetonitrile precipitation were investigated separately, and acetonitrile precipitation was selected because it excludes endogenous interference and has a higher extraction rate than methanol. Meanwhile, Waters ACQUITY UPLC BEH C18 (50 mm × 2.1 mm, 1.7 μm) and Waters ACQUITY UPLC HSS T3 columns (50 mm × 2.1 mm, 1.8 μm) were compared, and the previous separation was better than the latter and could fulfill the requirements of instrumental analysis.

### HDIs

Herbal medicines have a long history of use worldwide. With the development of complementary and alternative medicines, interactions between herbs and drugs are of increasing concern. There are two types of HDIs: a beneficial interaction that improves therapeutic outcomes and minimizes drug toxicity. Conversely, negative interactions may lead to unwanted clinical consequences, especially for drugs with low therapeutic indices (Zhou et al., 2021). HDIs may decrease efficiency, increase toxicity, and interfere with drug absorption and disposal processes due to interference from their pharmacological or pharmacokinetic effects (Cheng et al., 2023). Approximately 25% of herbal supplement users frequently take prescription drugs, which increases the likelihood of HDIs. The most commonly reported HDIs include modulation of drug-metabolizing enzymes and transporter proteins by herbal components and pharmacokinetic changes caused by co-administration of drugs as victims (Choi and Song, 2021).

CYP450 enzymes, which are essential for drug metabolism, are directly related to drug interactions. When ingested drugs have CYP activity, a possible interaction may occur that causes a variety of reactions, such as inhibition or induction of CYP activity (Dores et al., 2023). Our previous HDIs research results showed the following: plumbagin inhibited the metabolism of tazemetostat and increased the plasma exposure of tazemetostat in rats (Li et al., 2022), Chaihu Shugan Pills could slow down the absorption of duloxetine and reduce the exposure of duloxetine and its metabolites in plasma, that is, induce the metabolism of duloxetine (Bi et al., 2022), Danzhi Xiaoyao pill could reduce the plasma exposure of venlafaxine and increase the concentration of its metabolites, meaning that Danzhi Xiaoyao pills could influence the pharmacokinetics of venlafaxine in beagle dogs (Zhu et al., 2019).

Ayesha Tanveer, Khalid Hussain, and others reported that silymarin A altered the AUC and Cmax of chlosartan in a genotype-dependent manner. When administered concomitantly at normal doses in both CYP2C9*1/*1 and CYP2C9*1/*3 genotypes, there was a minor pharmacokinetic interaction between silymarin A and losartan. However, a moderate interaction was found in the CYP2C9*1/*2 genotype (Tanveer et al., 2022).

Cilofexor is a selective, non-steroidal FXR agonist that is metabolized by CYP2C8, 2C9 (Trauner et al., 2019). Meanwhile silybinin can inhibit CYP1A2, 2B6, 2C8, 2C9, 2C19, 2D6, and 3A4 (Xie et al., 2019). Therefore, pharmacokinetic HDIs based on CYP450 may occur. The results of this study showed that when cilofexor was used in combination with silybinin, the main pharmacokinetic parameters of cilofexor changed, Cmax, AUC_(0-t)_, and AUC_(0-∞)_ increased, t_1/2_ prolonged, and CL and Vd decreased. The results showed that silybinin could slow down cilofexor metabolism and increase plasma exposure. Therefore, when silybinin and cilofexor are used together clinically, cilofexor dosage should be adjusted to ensure efficacy and avoid adverse reactions.

The increasing consumption of various herbs and supplements raises serious health concerns. Owing to an insufficient understanding of the human development index, consuming these products at the same time may have harmful effects, and in extreme cases may even lead to fatal consequences. Therefore, it is necessary to increase the use of herbs/supplements, so that healthcare professionals can prescribe and counsel patients on appropriate and optimal treatment interventions, and enable patients to obtain and share validated and reliable information about the use of herbs/supplements (Dores et al., 2023). In the future, computer-aided or artificial intelligence-guided prediction of a wide range of HDIs will not only focus on the occurrence of toxicity but also on the changes in efficacy caused by different factors (such as synergistic and antagonistic effects), which may help promote the evaluation and interpretation of HDIs (Choi and Chin, 2020).

## Conclusion

In the present study, for the first time, a novel UPLC-MS/MS approach was successfully performed and applied to measure cilofexor in beagle dog plasma. Silybinin can inhibit cilofexor metabolism in beagle dogs, thereby affecting the pharmacokinetic parameters of cilofexor and increasing plasma exposure to cilofexor.

## Data Availability

The original contributions presented in the study are included in the article/Supplementary Material, further inquiries can be directed to the corresponding author.
